# A Curva Volume-Tempo Obtida pela Ecocardiografia Tridimensional na Cardiomiopatia Chagásica: Análise do Mecanismo das Adaptações Hemodinâmicas

**DOI:** 10.36660/abc.20201308

**Published:** 2022-04-25

**Authors:** Airandes de Sousa Pinto, Maria Carmo Pereira Nunes, Carlos Alberto Rodrigues, Bráulio Muzzi Ribeiro de Oliveira, João da Rocha Medrado, Timothy C. Tan, Manoel Otavio da Costa Rocha

**Affiliations:** 1 Programa de Pós-Graduação em Ciências da Saúde e Medicina Tropical Faculdade de Medicina Universidade Federal de Minas Gerais Belo Horizonte MG Brasil Programa de Pós-Graduação em Ciências da Saúde e Medicina Tropical da Faculdade de Medicina da Universidade Federal de Minas Gerais, Belo Horizonte, MG – Brasil; 2 Universidade Estadual de Feira de Santana Feira de Santana BA Brasil Universidade Estadual de Feira de Santana, Feira de Santana, BA – Brasil; 3 Universidade Federal de Minas Gerais Belo Horizonte MG Brasil Universidade Federal de Minas Gerais, Belo Horizonte, MG – Brasil; 4 Instituto de Previdência dos Servidores do Estado de Minas Gerais Belo Horizonte MG Brasil Instituto de Previdência dos Servidores do Estado de Minas Gerais – IPSEMG, Belo Horizonte, MG – Brasil; 5 University of Western Sydney Department of Cardiology Penrith South New South Wales Austrália University of Western Sydney – Department of Cardiology, Penrith South, New South Wales – Austrália

**Keywords:** Ecocardiografia Tridimensional, Fibrilação Atrial, Volume ejetado, Cardiopatia Chagásica, Lei de Frank-Starling

## Abstract

**Fundamento:**

A ecocardiografia tridimensional (ECO 3D) permite a geração de uma curva volume-tempo representativa das alterações no volume ventricular esquerdo (VE) ao longo de todo o ciclo cardíaco.

**Objetivo:**

O presente estudo tem como objetivo demonstrar as adaptações hemodinâmicas presentes na cardiomiopatia chagásica (CC) por meio das medidas de volume e fluxo obtidas pela curva volume-tempo por ECO 3D.

**Métodos:**

Vinte pacientes com CC e 15 indivíduos saudáveis foram incluídos prospectivamente em um estudo de desenho transversal. Realizou-se ECO 3D em todos os indivíduos e as curvas volume-tempo do VE foram geradas. O fluxo foi obtido pela primeira derivada da curva volume-tempo por meio do software MATLAB. A significância estatística foi definida com p<0,05.

**Resultados:**

Embora os pacientes com CC tivessem menor fração de ejeção do VE em comparação com o grupo controle (29,8±7,5 vs. 57,7±6,1, p<0,001), o volume (61,5±25,2 vs. 53,8±21,0, p=0,364) e o fluxo de ejeção máximo durante a sístole (-360,3±147,5 vs. -305,6±126,0, p = 0,231) mostraram-se semelhantes entre os grupos. Da mesma forma, o fluxo máximo na fase de enchimento inicial e durante a contração atrial mostrou-se semelhante entre os grupos. Um aumento na pré-carga expressa pelo volume diastólico final do VE (204,8±79,4 vs. 93,0±32,6), p<0,001) pode manter o fluxo e o volume ejetado semelhantes aos dos controles.

**Conclusão:**

Com uma ferramenta não invasiva, demonstramos que o aumento no volume diastólico final do VE pode ser o principal mecanismo de adaptação que mantém o fluxo e o volume ejetado no cenário de disfunção sistólica ventricular esquerda severa.

## Introdução

Os métodos atuais de ecocardiografia bidimensional (2D) para avaliação do volume ventricular esquerdo (VE) são limitados pela variabilidade inter-observador e por premissas geométricas.^1^ O advento da ecocardiografia tridimensional (ECO 3D) permitiu que os volumes ventriculares fossem avaliados sem o uso de quaisquer premissas geométricas, permitindo a geração de uma curva volume-tempo representativa das alterações no volume do VE ao longo de todo o ciclo cardíaco, estando, portanto, muito menos sujeitos à variabilidade do observador devido à detecção semiautomática das bordas do VE.^2^ No entanto, atualmente, o ECO 3D vir usado para a avaliação morfológica das estruturas cardíacas, a avaliação hemodinâmica ainda é realizada por meio de variáveis ecocardiográficas 2D, incluindo dimensão e velocidade na equação da continuidade. Embora as medidas uniplanares das dimensões VE sejam rotineiramente usadas para avaliar o aumento da câmara cardíaca, as medidas de volume 3D representam melhor a dilatação geral da câmara.^1^ Além disso, as medidas do fluxo instantâneo dentro de uma câmara cardíaca podem ser obtidas usando dados da primeira derivada das curvas de volume.

Esta abordagem não invasiva para a caracterização da dilatação das câmaras cardíacas não foi estudada em pacientes com cardiomiopatia chagásica. Portanto, este estudo tem como objetivo demonstrar as adaptações hemodinâmicas presentes na cardiomiopatia chagásica por meio das medidas de volume e fluxo obtidas pela curva volume-tempo por meio da ecocardiografia 3D.

## Métodos

**Figura 1:**
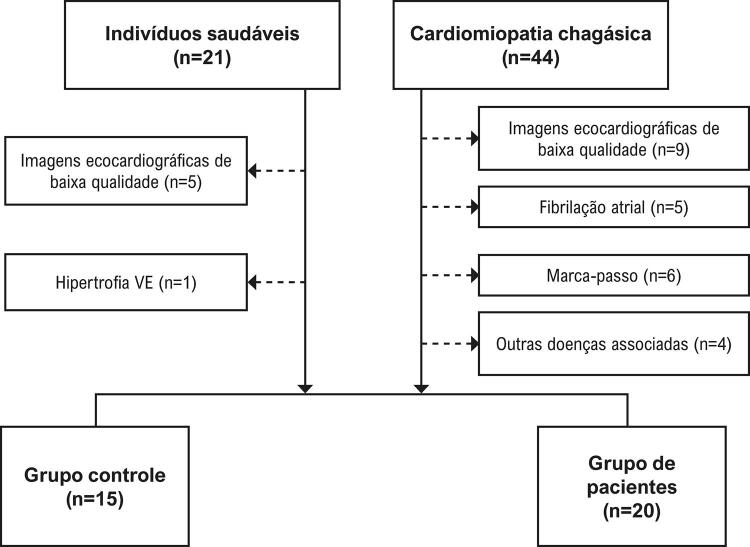
– Fluxograma do estudo.

Um total de 44 pacientes com cardiomiopatia chagásica foram inicialmente recrutados para o estudo. Foram excluídos pacientes com hipertensão arterial, fibrilação atrial, cardiopatia valvar, cardiopatia congênita, pericardiomiopatia e portadores de marca-passo. Com base nesses critérios de exclusão, 24 pacientes foram excluídos e 20 pacientes foram incluídos no estudo (fluxograma do estudo, Figura 1 ). Os participantes do grupo controle não apresentavam histórico clínica de doença cardiovascular. Os exames clínico e ecocardiográfico estavam normais.

Definiu-se cardiomiopatia chagásica como presença de fração de ejeção VE menor ou igual a 54% e diâmetro diastólico final VE maior que 56 mm.

**Figura 2:**
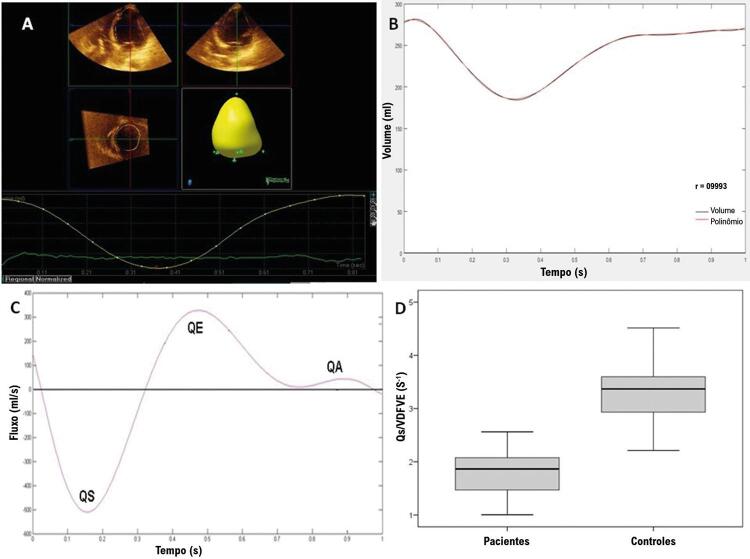
– A) Curva de volume do ventrículo esquerdo gerada pelo software Qlab em paciente com cardiomiopatia chagásica. B) Representação da curva de volume do ventrículo esquerdo, em preto, gerada pelo software Qlab e o intervalo do polinômio gerado pelo software MATLAB, em vermelho. C) Curva de fluxo obtida em paciente com cardiomiopatia chagásica durante o ciclo cardíaco. Valores negativos ocorrem durante a sístole e valores positivos durante a diástole. QS= Fluxo sistólico máximo absoluto, QE= fluxo de pico durante o enchimento ventricular esquerdo precoce. QA = pico de fluxo durante a contração atrial. D) Boxplot do valor absoluto do volume diastólico final do QS/VE de acordo com o grupo de estudo.

O estudo ecocardiográfico foi realizado por um único examinador, utilizando-se ecocardiógrafo IE 33-Philips de acordo com o protocolo da Sociedade Americana de Ecocardiografia.^3^ Realizou-se ecocardiografia tridimensional em todos os participantes com um transdutor X3-1. As curvas volume-tempo do ventrículo esquerdo foram geradas pelo software próprio Qlab ( Figura 2 , A). Essas curvas geraram o volume diastólico final ventricular esquerdo, o volume sistólico final ventricular esquerdo e o volume sistólico. A curva de volume foi criada em intervalos de cerca de 03 ms. O software MATLAB versão R2017a gerou um polinômio ajustado para a curva de volume ventricular esquerda ( Figura 2 , B). A correlação entre as curvas de volume geradas pelo Qlab e o polinômio obtido pelo Mathlab apresentou r≥0,99 em todos os pacientes.

Os valores de fluxo durante o ciclo cardíaco ( Figura 2 , C) foram obtidos pela primeira derivada do polinômio representativo da curva de volume.

Para nossa análise, utilizamos o fluxo máximo durante a sístole, enchimento precoce e contração atrial ( Figura 2 , C). Além disso, calculamos a razão entre o fluxo sistólico máximo e volume diastólico final ventricular esquerdo (QS/VDFVE) ( Figura 2 , D).

### Análise estatística

O estudo foi projetado para atingir 95% de poder para detectar uma redução de 50% na razão entre o pico do fluxo sistólico instantâneo (QS) e o volume diastólico final VE em pacientes com cardiomiopatia chagásica em comparação com o grupo controle com base nos valores obtidos por Marshall et al. (n1=12, n2=10, média x1=3,4 seg^-1^ e x2 = 1,22 seg^-1^).4 Portanto, considerando um erro alfa de 0,05 e uma razão paciente: controle de 1, obteve-se uma amostra de 3 pacientes e 3 controles. Para os cálculos, utilizou-se o software G Power versão 3.1.

Utilizou-se o teste do qui-quadrado para comparar as variáveis categóricas entre os grupos. As variáveis contínuas com distribuição normal foram expressas como média±desvio padrão ou como mediana ou intervalo interquartil se apresentassem distribuição não normal. Utilizou-se o teste de Shapiro-Wilk para avaliar a normalidade das variáveis.

Utilizou-se o teste t de Student não pareado para comparar variáveis contínuas com distribuição normal e o teste de Mann-Whitney para comparar variáveis com distribuição não normal entre os grupos.

As correlações foram realizadas pelo método de Pearson. A significância estatística foi definida como p<0,05. Todas as análises foram realizadas utilizando o software SPSS versão 15.0 (SPSS Inc., Chicago, IL).

O Comitê de Ética da Universidade Federal de Minas Gerais (CAAE:48354315.8.3001.5091) aprovou o estudo, e o consentimento informado por escrito foi obtido de todos os pacientes.

## Resultados

Vinte pacientes com CC, idade média de 45±12, 55% do sexo masculino, foram comparados com 15 controles saudáveis pareados por sexo e idade. Não houve diferença de sexo entre pacientes e controles. As características ecocardiográficas da população estudada encontram-se na tabela 1 . A maioria dos pacientes (70%) apresentava dispneia aos esforços, em tratamento para insuficiência cardíaca, principalmente com inibidores da enzima conversora da angiotensina e betabloqueadores ( Tabela 2 ).

**Tabela 1:** Características ecocardiográficas da população estudada

Variável*	Cardiomiopatia chagásica (n=20)	Controles (n=15)	Valor de p
Diâmetro diastólico final VE (mm)	68,4±9,2	46,6±4,2	<0,001
Diâmetro sistólico final VE (mm)	56,1±10,8	30,1±3,7	<0,001
Volume diastólico final VE (mL)	204,8±79,4	93,0±32,6	<0,001
Volume sistólico final VE (mL)	143,3±60,8	39,2±13,6	<0,001
Volume sistólico (mL)	61,5±25,2	53,8±21,0	0,364
Fração de ejeção VE 3D (%)	29,8±7,5	57,7±6,1	<0,001
QS (mL/s)	- 360,3±147,5	-305,6±126,0	0,231
QS/volume diastólico final VE (s-1)	1,80±0,40	3,28±0,64	<0,001
QE (mL/s)	270,4±135,3	201,9±61,5	0,104
QA (mL/s)	134,4±88,1	109,1±37,8	0,623
QE/QA	2,2±1,3	1,8±0,5	0,382
Velocidade de pico mitral E (m/s)	81,0±30,2	81,9±19,5	0,921
Tempo de desaceleração (ms)	166,5 (79)	190,0 (38)	0,290
Velocidade de pico mitral A (m/s)	51,2±24,5	55,4±15,6	0,583
Razão E/A mitral	1,9±1,1	1,6±0,6	0,404
Razão E/e’	15,2±9,3	7,6±1,7	0,002

*Os dados são expressos como média±desvio padrão ou mediana (intervalo interquartil). VE: ventricular esquerdo; QS: pico de fluxo sistólico instantâneo; QE: pico de fluxo durante o enchimento ventricular esquerdo inicial; QA: pico de fluxo durante a contração atrial.*

**Tabela 2:** Medicamentos usados pelos 20 pacientes com cardiomiopatia chagásica crônica dilatada

Medicações	Número de pacientes (%)
Diuréticos	19 (95)
Espironolactona	5 (25)
Inibidores da enzima conversora da angiotensina	16 (80)
Antagonistas dos receptores de angiotensina	3 (15)
Digoxina	13 (65)
Amiodarona	6 (30)
Terapia anticoagulante	8 (40)
Betabloqueadores	17 (85)
Aspirina	1 (5)

A frequência cardíaca (batimentos por minuto) foi semelhante entre a cardiomiopatia chagásica e o grupo controle — 62,4±10,2 vs. 66,1±11,0, p=0,3, respectivamente.

Os pacientes com CC apresentaram maiores volumes diastólico e sistólico final de VE e menor fração de ejeção VE, em comparação ao grupo controle. No entanto, o volume ejetado e o fluxo de ejeção máximo durante a sístole (QS) foram semelhantes entre os grupos. Houve uma forte correlação entre o QS e o volume sistólico: r=0,91, p<0,001.

O grupo CC apresentou uma razão QS/volume diastólico final do VE menor em comparação com os controles ( Figura 2 , D). A razão QS/volume diastólico final do VE apresentou forte correlação com a fração de ejeção: r=0,89, p<0,001.

A avaliação por Doppler da velocidade mitral não mostrou nenhuma diferença em E, A, razão E/A e tempo de desaceleração da onda E. Como esperado, os pacientes com CC apresentaram aumento da pré-carga em comparação ao grupo controle, demonstrado pelo aumento do volume diastólico final do VE e da razão E/e’.

O fluxo máximo na fase de enchimento passivo e precoce (QE) e durante a contração atrial (QA) se mostrou semelhante entre pacientes e controles.

## Discussão

Em nosso estudo, avaliamos as adaptações hemodinâmicas do VE na CC por meio de curvas de volume e fluxo por ecocardiografia 3D em comparação a um grupo controle. Embora os pacientes com CC tivessem função sistólica VE severa com fração de ejeção de 30%, o volume ejetado foi semelhante ao grupo controle. Essa discrepância pode ser explicada pelos mecanismos adaptativos que ocorrem na disfunção sistólica crônica do VE.^5 , 6^ O ventrículo com baixa fração de ejeção, mas com volume diastólico final elevado, ejeta a mesma quantidade de sangue que um ventrículo com volume diastólico final e fração de ejeção normais.^7^ Isso se deve à preservação do mecanismo de Frank-Starling na CC em repouso, o que está de acordo com os achados de Holubasch et al.^5^

A ecocardiografia tridimensional permite a medição não invasiva da pré-carga, apresentando alta precisão. O volume diastólico final do VE é a melhor representação da pré-carga, que expressa o grau de estiramento do miocárdio antes da contração. As limitações na avaliação precisa do volume ventricular por métodos ecocardiográficos padrão levam ao uso de pressões de enchimento ventricular como medida substituta da pré-carga. No entanto, a relação entre as pressões de enchimento e o volume ventricular não é linear, dependendo da complacência da câmara cardíaca esquerda.^8^

A curva volume-tempo pela ecocardiografia 3D também fornece informações para o cálculo do fluxo em qualquer estágio do ciclo cardíaco. Em nosso estudo, o fluxo foi obtido por interpolação polinomial. A interpolação polinomial é um método preciso de baixa complexidade que permite medir a variação de qualquer curva derivável. Recentemente, usamos essa ferramenta para realizar uma análise da taxa de crescimento da Covid-19.^9 , 10^

O fluxo máximo de ejeção (QS) se mostrou semelhante entre os grupos, não refletindo a função sistólica do ventrículo esquerdo. A forte correlação entre o QS absoluto e o volume ejetado sugere que o mesmo mecanismo que normalizou o volume sistólico (VS) competiu pela normalização do QS. Portanto, o QS/volume diastólico final do VE retira o efeito da dilatação ventricular esquerda, que é o aumento da pré-carga, e produz uma variável que permite avaliar a função sistólica global do VE. De fato, em nosso estudo, o QS absoluto/volume diastólico final do VE foi menor nos pacientes que tinham CC do que nos controles normais, o que está de acordo com os achados de outros autores.^4 , 11 , 12^

Esse artifício é o mesmo usado para calcular a fração de ejeção. Dividindo-se o VS pelo volume diastólico final do ventrículo esquerdo, o resultado é mais do que uma porcentagem do volume final do ventrículo esquerdo que é ejetado. A proporção representa a normalização do volume sistólico pelo representante da pré-carga: o volume final do VE. Como a pré-carga é um dos determinantes da função sistólica, isso pode explicar a importância prognóstica da fração de ejeção nas cardiomiopatias.

Da mesma forma, Hammersmeister et al.,^11^ validaram um método de avaliação do volume e fluxo VE em 1974, em diversas doenças cardiovasculares, por cateterismo cardíaco.^11^ O volume ventricular foi calculado por ventriculografia na frequência de 60 quadros/s, pelo método área-comprimento. O fluxo foi obtido pela primeira derivada do polinômio que se aproximou da curva de volume. No entanto, esse método é limitado devido à sua natureza invasiva. Por outro lado, em nosso estudo, obtivemos a curva de volume VE durante o ciclo cardíaco com frequência três vezes maior do que método semelhante ao descrito por Hammermeister et al.,^11^ Além disso, encontramos uma forte correlação entre o polinômio e a curva de volume VE, permitindo o cálculo do fluxo com grande precisão.

A ausência de diferença entre os valores do fluxo diastólico entre os grupos também foi observada por Hammermeister et al.,^13^ O comportamento em “U” dessas variáveis frente à piora da função diastólica explica esses resultados, conforme observado por Ohno et al.,^6^ em um estudo experimental.^6^ Apesar disso, a razão E/e’ foi maior no grupo com CC do que no grupo controle, o que está de acordo com os achados de Oliveira et al.,^14^ que observaram que essa variável foi um preditor independente para nível elevado de peptídeo natriurético tipo B (BNP) na CC.^14^

A ecocardiografia tridimensional permite revisitar os estudos experimentais do início do século passado, quando o mecanismo de Frank-Starling foi descrito e os fatores mecânicos relacionados ao volume e os fatores mecânicos relacionados ao volume ejetado, reconhecido na época como uma medida da função cardíaca, foram estudados.^15^

Este estudo teve as seguintes limitações: a função diastólica do ventrículo esquerdo não foi classificada, mas foram tomados os parâmetros para avaliar a função diastólica. Os valores normais para o QS/volume diastólico final VE basearam-se nos controles, que podem não ser os valores de referência. Finalmente, a importância clínica e as implicações prognósticas desses achados ainda não são totalmente conhecidas. No entanto, este estudo teve como objetivo demonstrar as adaptações hemodinâmicas presentes na cardiomiopatia chagásica por meio das medidas de volume e fluxo obtidas pela curva volume-tempo.

## Conclusões

Nosso estudo mostra que o fluxo sistólico instantâneo e o volume ejetado foram semelhantes entre pacientes com disfunção ventricular grave devido a CC e controles saudáveis. Utilizando uma ferramenta não invasiva pela primeira vez na CC, demonstramos que o aumento no volume diastólico final VE, que é uma medida da pré-carga ventricular, é o principal mecanismo de adaptação que mantém o fluxo e o volume ejetado no cenário de disfunção sistólica severa. O QS/volume diastólico final VE, no presente estudo, mostrou-se representativo da função sistólica global do ventrículo esquerdo, cuja utilidade e valor prognóstico devem ser estudados em pesquisas cuja utilidade e valor prognóstico devem ser estudados em pesquisas posteriores.
