# Supplementary material for: A Curva Volume-Tempo Obtida pela Ecocardiografia Tridimensional na Cardiomiopatia Chagásica: Análise do Mecanismo das Adaptações Hemodinâmicas
Source: Arq Bras Cardiol. 2022 Apr 25;118(6):1099–105. [Article in Portuguese] doi: 10.36660/abc.20201308 (PMC9345165; doi:10.36660/abc.20201308)
Supplement: Translation

# Translation: The Volume-Time Curve by Three-Dimensional Echocardiography in Chagas Cardiomyopathy: Insights into the Mechanism of Hemodynamic Adaptations

**Authors:** Airandes de Sousa Pinto, Maria Carmo Pereira Nunes, Carlos Alberto Rodrigues, Bráulio Muzzi Ribeiro de Oliveira, João da Rocha Medrado, Timothy C. Tan, Manoel Otavio da Costa Rocha

**Affiliations:** 1 Brasil Programa de Pós-Graduação em Ciências da Saúde e Medicina Tropical da Faculdade de Medicina da Universidade Federal de Minas Gerais, Belo Horizonte, MG – Brasil; 2 Brasil Universidade Estadual de Feira de Santana, Feira de Santana, BA – Brasil; 3 Brasil Universidade Federal de Minas Gerais, Belo Horizonte, MG – Brasil; 4 Brasil Instituto de Previdência dos Servidores do Estado de Minas Gerais – IPSEMG, Belo Horizonte, MG – Brasil; 5 Austrália University of Western Sydney – Department of Cardiology, Penrith South, New South Wales – Austrália

**Keywords:** Echocardiography, Three Dimensional Echocardiogram, Atrial Fibrillation, Stroke Volume, Chagas Cardiomyopathy, Frank-Starling Law

## Abstract

**Background:**

Three-dimensional echocardiography (3D ECHO) allows the generation of a volume-time curve representative of changes in the left ventricular (LV) volume throughout the entire cardiac cycle.

**Objective:**

This study aims to demonstrate the hemodynamic adaptations present in Chagas cardiomyopathy (CC) by means of the volume and flow measurements obtained by the volume-time curve by 3D ECHO.

**Methods:**

Twenty patients with CC and 15 healthy subjects were prospectively enrolled in a cross-sectional design study. 3D ECHO was performed in all subjects and the volume over time curves of the LV was generated. The flow was obtained by the first derivative of the volume-time curve using the software MATLAB. Statistical significance was set at p<0.05.

**Results:**

Although CC patients had lower LV ejection fraction compared to the control group (29.8±7.5 vs. 57.7±6.1, p<0.001), stroke volume (61.5±25.2 vs. 53.8±21.0, p=0.364) and maximum ejection flow during systole (-360.3±147.5 vs. -305.6±126.0, p=0.231) were similar between the groups. Likewise, the maximum flow in the early diastolic filling phase and during atrial contraction was similar between groups. An increase in preload expressed by LV end diastolic volume (204.8±79.4 vs. 93.0±32.6), p<0.001) may maintain the flow and stroke volumes similar to the controls.

**Conclusion:**

Using a non-invasive tool, we demonstrated that an increase in LV end-diastolic volume may be the main adaptation mechanism that maintains the flow and stroke volumes in the setting of severe LV systolic dysfunction.

## Introduction

Current two-dimensional (2D) echocardiography methods for the assessment of left ventricular (LV) volume are limited by observer variability, and geometric assumptions.^1^ The advent of three-dimensional echocardiography (3D ECHO) allowed ventricular volumes assessed without using any geometric assumptions, allowing the generation of a volume-time curve representative of changes in LV volume throughout the entire

cardiac cycle, thus much less subject to observer variability due to the semiautomated detection of LV edges.^2^ However, currently 3D ECHO has been used for morphological evaluation of cardiac structures, but hemodynamic evaluation is still performed using 2D echocardiographic variables, including dimension and velocity in the continuity equation. Although single plane measurements of LV size are routinely used to evaluate cardiac chamber enlargement, 3D volume measurements best represent overall chamber dilatation.^1^ In addition, measurements of instantaneous flow within a cardiac chamber can be obtained using data from the first derivative of volume curves.

This non-invasive approach for characterization of cardiac chamber dilatation has not been studied in patients with Chagas cardiomyopathy. Therefore, this study aims to demonstrate the hemodynamic adaptations present in Chagas cardiomyopathy using the measures of volume and flow obtained by volume-time curve using 3D echocardiography.

## Methods

A total of 44 patients presenting Chagas cardiomyopathy were initially recruited for the study. Patients with arterial hypertension, atrial fibrillation, valvular heart disease, congenital heart disease, pericardiomyopathy, and those who had pacemakers were excluded. Based on these exclusion criteria, 24 patients were excluded and 20 patients were included in the study (study flowchart, Figure 1 ). The individuals in the control group had no clinical history of cardiovascular disease. Clinical and echocardiographic examinations were normal.

**Figure 1:** – Study population flow chart.

Chagas cardiomyopathy was defined as the presence of LV ejection fraction smaller than or equal to 54% and LV end-diastolic diameter greater than 56 mm.

The echocardiographic study was performed by a single examiner, using a IE 33-Philips echocardiograph according to the protocol of the American Society of Echocardiography.^3^ Three-dimensional echocardiography was performed in all subjects using a X3-1 transducer. The volume-time curves of the left ventricle were generated by proprietary software Qlab ( Figure 2 , A). These curves yielded left ventricular end-diastolic volume, left ventricular end-systolic volume and stroke volume. The volume curve was generated at intervals of around 3 ms. The software MATLAB version R2017a generated a polynomial adjusted to the left ventricular volume curve ( Figure 2 , B). The correlation between the volume curves generated by Qlab and the polynomial obtained by Mathlab presented r≥0.99 in all patients.

**Figure 2:** – A) Left ventricular volume curve generated by Qlab software in a patient with Chagas cardiomyopathy. B) Representation of the left ventricular volume curve, in black, generated by the Qlab Software and the interval of the polynomial generated by the MATLAB software, in red. C) Flow curve obtained in a patient with Chagas cardiomyopathy during the cardiac cycle. Negative values occur during systole and positive values during diastole. QS= Absolute maximum systolic flow, QE= peak flow during early left ventricular filling. QA = peak flow during atrial contraction. D) Boxplot of the absolute value of the QS/LV end-diastolic volume according to the study group.

The flow values during cardiac cycle ( Figure 2 , C) were obtained by the first derivative of the representative polynomial of the volume curve.

For our analysis, we used the maximum flow during systole, early filling and atrial contraction ( Figure 2 , C). In addition, we calculated the maximum flow systole divided by left ventricular end-diastolic volume (QS/LVEDV) ( Figure 2 , D).

### Statistical analysis

This study was designed to achieve 95% power to detect a 50% reduction in the ratio between peak instantaneous systolic flow (QS) and LV end-diastolic volume in patients with Chagas cardiomyopathy compared to the control group based on the values obtained by Marshall et al. (n1=12, n2=10, mean x1=3.4 sec^-1^and x2=1.22 sec^-1^).^4^ Therefore, considering an alpha error of 0.05 and a patient:control ratio of 1, a sample of 3 patients and 3 controls was obtained. For the calculations, the G Power software version 3.1 was used.

Chi-square test was used to compare the categorical variables between the groups. The continuous variables with normal distribution were expressed as mean±standard deviation or as median or interquartile range if they presented a non-normal distribution. We used the Shapiro-Wilk test to assess the normality of the variables.

Unpaired Student’s t test was used to compare continuous variables with normal distribution, and the Mann-Whitney test was used to compare variables with non-normal distribution between the groups.

The correlations were performed using the Pearson method. Statistical significance was set at p<0.05. All analyzes were performed using the software SPSS version 15.0 (SPSS, Inc., Chicago, IL).

This study was approved by the Research Ethics Committee of Universidade Federal de Minas Gerais (CAAE:48354315.8.3001.5091) and written informed consent was obtained from all patients.

## Results

Twenty patients with CC, mean age 45±12, 55% males, were compared with 15 sex- and age-matched healthy controls. There was no sex difference between patients and controls. The echocardiographic characteristics of the study population are shown in Table 1 . The majority the patients (70%) had exertional dyspnea, on treatment for heart failure, mainly using angiotensin-converting enzyme inhibitors and beta-blockers ( Table 2 ).

**Table 1:** Echocardiographic characteristics of the study population

Variable *	Chagas cardiomyopathy (n=20)	Controls (n=15)	p value
LV end-diastolic diameter (mm)	68.4±9.2	46.6±4.2	<0.001
LV end-systolic diameter (mm)	56.1±10.8	30.1±3.7	<0.001
LV end-diastolic volume (mL)	204.8±79.4	93.0±32.6	<0.001
LV end-systolic volume (mL)	143.3±60.8	39.2±13.6	<0.001
Stroke volume (mL)	61.5±25.2	53.8±21.0	0.364
3D LV ejection fraction (%)	29.8±7.5	57.7±6.1	<0.001
QS (mL/s)	- 360.3±147.5	-305.6±126.0	0.231
QS/LV end-diastolic volume (s-1)	1.80±0.40	3.28±0.64	<0.001
QE (mL/s)	270.4±135.3	201.9±61.5	0.104
QA (mL/s)	134.4±88.1	109.1±37.8	0.623
QE/QA	2.2±1.3	1.8±0.5	0.382
Mitral peak E velocity (m/s)	81.0±30.2	81.9±19.5	0.921
Deceleration time (ms)	166.5 (79)	190.0 (38)	0.290
Mitral peak A velocity (m/s)	51.2±24.5	55.4±15.6	0.583
Mitral E/A ratio	1.9±1.1	1.6±0.6	0.404
E/e’ ratio	15.2±9.3	7.6±1.7	0.002

*Data are expressed as mean±standard deviation, or median (interquartile range). LV: left ventricular; QS:peak instantaneous systolic flow; QE: peak flow during early left ventricular filling; QA: peak flow during atrial contraction.*

**Table 2:** Medications used by the 20 patients with chronic dilated Chagas cardiomyopathy

Medications	Number of patients (%)
Diuretics	19 (95)
Spironolactone	5 (25)
Angiotensin-converting enzyme inhibitors	16 (80)
Angiotensin receptor antagonists	3 (15)
Digoxin	13 (65)
Amiodarone	6 (30)
Anticoagulant therapy	8 (40)
Beta-blockers	17 (85)
Aspirin	1 (5)

Heart rate (beats per minute) was similar between the Chagas cardiomyopathy and the control group — 62.4±10.2 vs. 66.1± 11.0, p=0.3, respectively.

The patients with CC had greater LV end-diastolic and end-systolic volumes, and lower LV ejection fraction, compared to the control group. However, stroke volume and maximum ejection flow during systole (QS) were similar between the groups. There was a strong correlation between QS and stroke volume: r=0.91, p<0.001.

The CC group had a lower QS/LV end-diastolic volume ratio compared with the controls ( Figure 2 , D). The QS/LV end-diastolic volume ratio presented a strong correlation with the ejection fraction: r=0.89, p<0.001.

Doppler evaluation of mitral velocity did not show any difference in E, A, E/A ratio and E wave deceleration time. As expected, the patients with CC showed an increase in preload compared with the control group, as demonstrated by an increased LV end-diastolic volume and E/e’ ratio.

The maximum flow in the early and passive filling phase (QE) and during atrial contraction (QA) was similar between patients and controls.

## Discussion

In our study, we evaluated the hemodynamic adaptations of the LV in CC using volume and flow curves by 3D echocardiography compared to a control group. Although the patients with CC had severe LV systolic function with ejection fraction of 30%, the stroke volumes were similar to controls. This discrepancy may be explained by the adaptive mechanisms that occur in chronic LV systolic dysfunction.^5 , 6^ The ventricle with low ejection fraction but with increased end-diastolic volume ejects the same amount of blood as a ventricle with normal end-diastolic volume and ejection fraction.^7^ This is due to preservation of the Frank-Starling mechanism in CC at rest, which is in agreement with the findings of Holubasch et al.^5^

Three-dimensional echocardiography allows non-invasive preload measurement with high accuracy. End-diastolic LV volume is the best representation of preload, which expresses the degree of myocardial stretch before contraction. Limitations in evaluating accurately ventricular volume by standard echocardiographic methods lead to used ventricular filling pressures as a surrogate measurement of preload. However, the relationship between filling pressures and ventricular volume is not linear, depending on the compliance of left-sided cardiac chamber.^8^

The volume-time curve by 3D echocardiography also provides information for calculating flow at any stage of the cardiac cycle. In our study, the flow was obtained by polynomial interpolation. Polynomial interpolation is an accurate low-complexity method that allows to measure the variation of any derivable curve. We recently used this tool to conduct a Covid-19 growth rate analysis.^9 , 10^

Maximal ejection flow (QS) was similar between the groups, which did not reflect left ventricular systolic function. The strong correlation between absolute QS and stroke volume suggests that the same mechanism that normalized the Stroke volume competed for the normalization of QS. Therefore, QS/LV end-diastolic volume withdraws the effect of left ventricular dilatation, which is increased preload, and derive a variable that allows assessing LV global systolic function. Indeed, in our study, absolute QS/LV end-diastolic volume was lower in those patients who had CC than in normal controls, which is in agreement with the findings of other authors.^4 , 11 , 12^

This artifice is the same used to calculate ejection fraction. By dividing the systolic volume (SV) by end-diastolic left ventricular volume, the result is more than a percentage of the final left ventricular volume that is ejected. The ratio represents the normalization of stroke volume by the representative of preload: LV end volume. Since preload is one of the determinants of systolic function, this may explain the prognostic importance of ejection fraction in cardiomyopathies.

Similarly, Hammersmeister et al.^11^ validated a method for assessing LV volume and flow in 1974, in several cardiovascular diseases, by cardiac catheterization.^11^ Ventricular volume was calculated by ventriculography at a frequency of 60 frames/s, using the area-length method. The flow was obtained by the first derivative of the polynomial that approached the volume curve. However, this method is limited due to its invasive nature. On the other hand, in our study, we obtained the LV volume curve during the cardiac cycle with a frequency three times greater than a similar method described by Hammermeister et al.^11^ In addition, we found a strong correlation between the polynomial and LV volume curve, allowing the calculation of flow with great accuracy.

The absence of difference between diastolic flow values between groups was also observed by Hammermeister et al.^13^ The “U” behavior of these variables considering diastolic function worsening explains these results, as observed by Ohno et al^6^ in an experimental study.^6^ Despite this, the E/e’ratio was higher in the group with CC than in the control group, which is in agreement with Oliveira et al.,^14^ who observed that this variable was an independent predictor for elevated brain natriuretic peptide (BNP) levels in CC.^14^

Three-dimensional echocardiography allows to revisit experimental studies from the beginning of the last century, when the Frank-Starling mechanism was described and the mechanical factors related to stroke volume, recognized at that time as a measure of cardiac function, were studied.^15^

This study had the following limitations: left ventricular diastolic function was not classified, but the parameters to assess diastolic function were taken. The normal values for QS/LV end-diastolic volume was based on the controls, which may not be the reference values. Finally, the clinical importance and prognostic implications of these findings are not fully known yet. However, our objective was to demonstrate the hemodynamic adaptations present in Chagas cardiomyopathy using the measures of volume and flow obtained by the volume-time curve.

## Conclusions

Our study shows that instantaneous systolic flow and stroke volume were similar between patients with severe ventricular dysfunction due to CC and healthy controls. Using a non-invasive tool for the first time in CC, we demonstrated that an increase in LV end-diastolic volume, which is a measure of ventricular preload, is the main adaptation mechanism that maintains the flow and stroke volumes in the setting of severe systolic dysfunction. QS/LV end-diastolic volume, in this study, was shown to be representative of left ventricular global systolic function, whose usefulness and prognostic value should be studied in later studies.
